# 3D Mammary Epithelial Cell Models: A Goldmine of DCIS Biomarkers and Morphogenetic Mechanisms

**DOI:** 10.3390/cancers11020130

**Published:** 2019-01-23

**Authors:** Stefano Rossetti, Nicoletta Sacchi

**Affiliations:** Department of Cancer Genetics and Genomics, Roswell Park Comprehensive Cancer Center, Buffalo, NY 14263, USA; stefano.rossetti@roswellpark.org

**Keywords:** ductal carcinoma in situ (DCIS), 3D HME1 models, protein-miRNA pairs, DCIS biomarkers and morphogenetic mechanisms

## Abstract

Breast ductal carcinoma in situ (DCIS) has been typically recognized by pathologists on the basis of aberrant mammary duct morphology. Thus, there are increasing efforts to detect DCIS biomarkers and druggable targets. In this study we focused on the molecular mechanism involving Annexin A8 (ANXA8), a Ca^2+^ and phospholipid binding protein, which is regulated by all-trans Retinoic Acid (RA), and it is highly expressed in breast DCIS tissue samples relative to atypical ductal hyperplasia, and normal breast tissue. Using a panel of human mammary epithelial HME1 cell lines that share a common protein signature, and develop in vitro three dimensional (3D) “DCIS-like” amorphous structures, we identified by bioinformatics analysis protein-miRNA pairs, potentially involved in mammary morphogenetic mechanisms, including the ANXA8 mechanism. HME1 cells with genetic mutations hampering the physiological RA regulation of the RA receptor alpha (RARA) transcriptional function, but retain the RARA function controlling the PI3KCA-AKT signaling, develop 3D “DCIS-like” amorphous structures with upregulated ANXA8. Consistently, ectopic ANXA8 expression, by affecting the RARA transcriptional function, induced HME1 DCIS-like amorphous acini expressing phosphorylated AKT (P-AKT). Apparently, a RA-RARA-ANXA8 feedback loop fosters a vicious circle of aberrant morphogenesis. Interestingly, a few miRNAs regulated by RA are predicted to target ANXA8 mRNA. These miRNAs are candidate components of the RA-RARA-ANXA8 mechanism, and their deregulation might induce DCIS initiation.

## 1. Introduction

Early breast cancer stages, such as ductal carcinoma in situ (DCIS), are characterized by confined breast lesions with aberrant ductal morphological features. When left untreated, these in situ lesions can become invasive [1,2,3]. Detecting and treating breast cancer at its earlier stages can greatly increase survivability. To identify early stage breast cancer and prevent disease progression, research efforts have been focused on the identification of DCIS biomarkers and druggable targets. However, despite these efforts, the heterogeneity of breast DCIS lesions has made it extremely challenging to identify biomarkers with high diagnostic and prognostic value [4] that can be used also in liquid biopsies [5]. In an attempt to identify biomarkers with high sensitivity and specificity, we set out to use an unconventional experimental approach based on three dimensional (3D) mammary epithelial cell models. When grown in basement membrane culture non tumorigenic mammary epithelial cells, including HME1 cells, form 3D structures with a lumen-enclosing epithelial monolayer of apicobasal polarized cells, which is typical of normal mammary ducts [1,6,7,8]. In contrast, HME1 cells carrying different genetic mutations- hereafter referred to as HME1 “DCIS-precursor” cell lines- develop 3D DCIS-like structures with a luminal space filled with proliferating cells and loss of apicobasal polarity. HME1“DCIS-precursor” cell lines when grown in 3D basement membrane culture evade the growth inhibitory and pro-apoptotic effects regulated by physiological Retinoic Acid (RA) via the RA receptor alpha (RARA) transcriptional function, which is indispensable for lumen formation [7,9,10,11,12], but retain the RARA function that enables the physiological RA induction of AKT signaling by the phosphatidylinositol-3 kinase catalytic (PI3KCA) subunit ([13] and references within). 

In this study we used a panel of HME1 “DCIS-precursor” cell lines including the following lines: HME1-RARA403, expressing a dominant negative RARA mutant lacking the RA binding domain that affects the RARA transcriptional function [14], but it does not affect the RARA function controlling the PI3K-AKT signaling pathway [13]; HME1-shERA [15] with downregulated Estrogen Receptor alpha (ERA), a transcriptional regulator of RARA [16,17]; HME1-shPER2 [15] with down regulated PERIOD 2, a circadian clock gene with tumor suppressor function in breast cancer [18] [19]; HME1*-*shMTG16 [20] with downregulation of MTG16, a tumor suppressor of breast cancer [21], HME1-MYC [22] overexpressing the MYC oncogene that regulates RARA [23].

Previous proteomics analysis of the HME1 “DCIS-precursor” cell lines let us identify a common protein signature with both upregulated and downregulated proteins [24]. This signature included Annexin 8 (ANXA8) and Annexin 2 (ANXA2), which are members of the Annexin family of Ca^2+^-binding proteins and present Retinoic Acid-Responsive Elements (RARE) in their regulatory regions [24]. 

**Figure 1:**
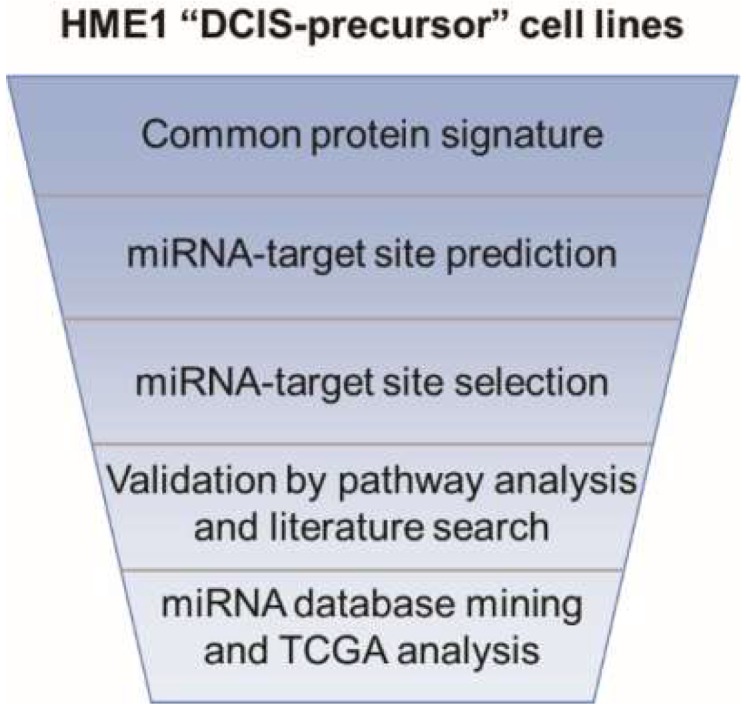
Scheme of the stepwise approach used to identify protein-miRNA pairs involved in human mammary morphogenetic mechanisms.

These findings attracted our attention because ANXA8 is upregulated in acute promyelocytic leukemia (APL) carrying PML-RARA, a dominant negative RARA mutant that inhibits the physiological RA regulation of wild type RARA transcriptional function [25]. ANXA8 is associated with a restricted signature of miRNAs that discriminates APL myeloblasts with dominant RARA fusion proteins from normal promyelocytes with wild type RARA [26]. According to the Cancer Genome Atlas (TCGA) breast cancer is not characterized by RARA fusion proteins. However, ANXA8 is significantly upregulated in breast DCIS relative to atypical ductal hyperplasia (ADH) and normal breast tissue, and it is also associated with clinical features of breast cancer progression (e.g., positive nodes, tumor stage, and tumor grade) [24]. Based on the common protein signature of HME1”DCIS-precursor” cell lines with mutations that affect RA-RARA signaling, we identified by a stepwise approach (Figure 1) a set of protein-miRNA pairs, including ANXA8-miRNA pairs. In the list of miRNAs, predicted to target ANXA8 mRNA 3’UTR, three miRNAs were encoded by genes with RA-responsive elements (RAREs) in their promoters [24,26]. 

Further, we used a 3D HME1-ANXA8^GFP^ model carrying a RARE-Green Fluorescent Protein (GFP) to assess the effects of ANXA8 upregulation on the spatial and temporal dynamics of the two RARA functions regulated by physiological RA in the course of 3D morphogenesis. These studies let us detect a RA-RARA-ANXA8 feedback loop that creates a vicious circle whereby P-AKT signaling fosters 3D aberrant morphogenesis.

## 2. Results 

### 2.1. From a Common Protein Signature Shared by HME1 “DCIS-Precursor” Cell Lines to a Subset of Potential Morphoregulatory miRNAs 

**Figure 2:**
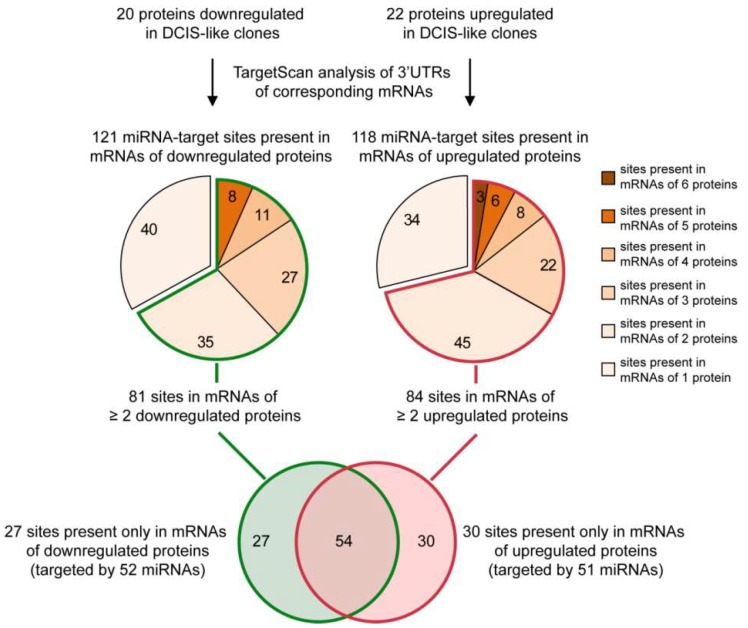
A protein-to-miRNA approach detects a miRNA signature shared by HME1“DCIS –precursor” cell lines. See Figure 3 and Figure 4 for details on miRNA-target sites present in the mRNAs of two or more downregulated or upregulated proteins.

Harnessing a common forty-two protein signature shared by HME1 “DCIS-precursor” cell lines with different mutations that we described previously [24], we set out to identify an in silico common miRNA signature. To this end we used the following stepwise approach. First, we analyzed the mRNA 3′UTR of 42 proteins differentially expressed in the HME1 “DCIS-precursor” cell lines vs. parental control HME1 cells by using TargetScan, a widely used algorithm that predicts miRNA-target sites based on their conservation among species [27]. Second, by focusing only on the most conserved miRNA-target sites (broadly conserved either in vertebrates or conserved only in mammals), we identified 121 sites in the mRNAs of 20 downregulated proteins, and 118 sites in the mRNAs of 22 upregulated proteins (Figure 2). 

Since each conserved site can be bound by one or more miRNAs, we found over 200 miRNAs predicted to target the mRNAs of the downregulated and upregulated proteins. Next, we grouped miRNA-target sites based on their frequency in the mRNAs of the proteins deregulated in the HME1 “DCIS-precursor” cell lines (Figure 2). We reasoned that if two or more upregulated (or downregulated) proteins have the same miRNA target site, it would be more likely that the miRNA(s) targeting this site are indeed deregulated.

**Figure 3:**
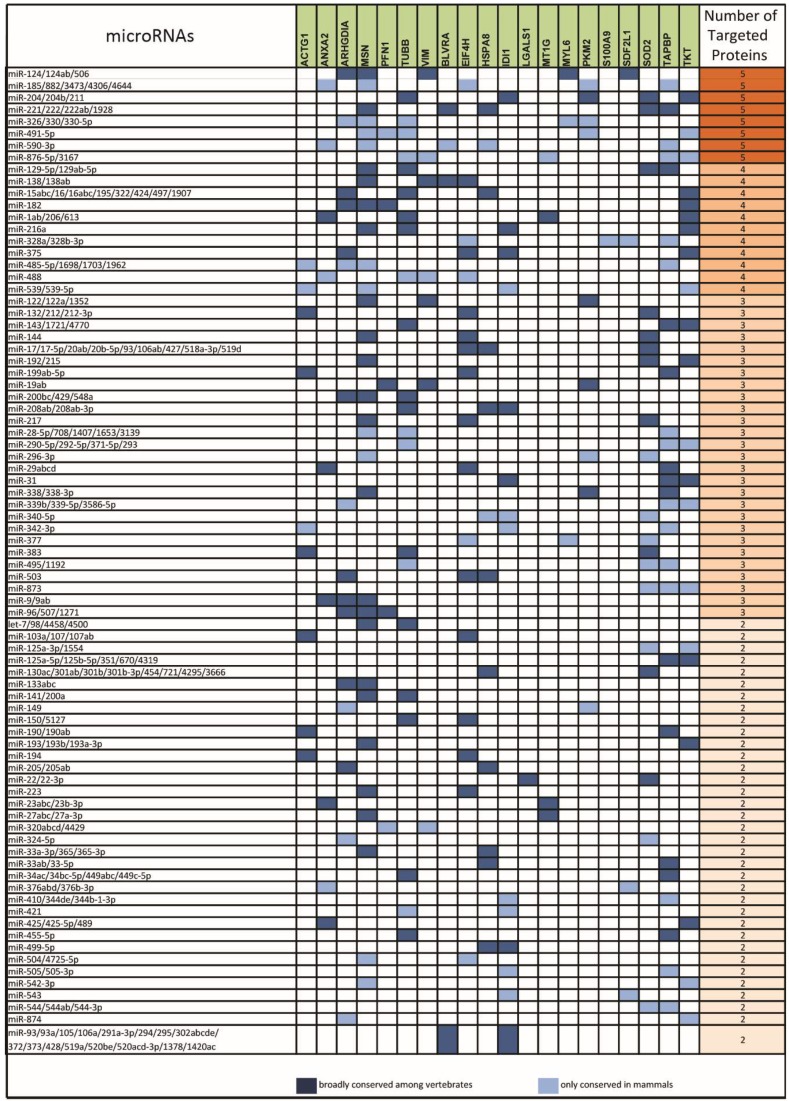
List of microRNAs (left column) targeting the sites present in the 3′UTR of mRNAs ofdownregulated proteins (middle column) in HME1 “DCIS-precursor “cell lines. Only target sites present in the 3′UTR of mRNAs of two, or more, downregulated proteins are shown (right column).

**Figure 4:**
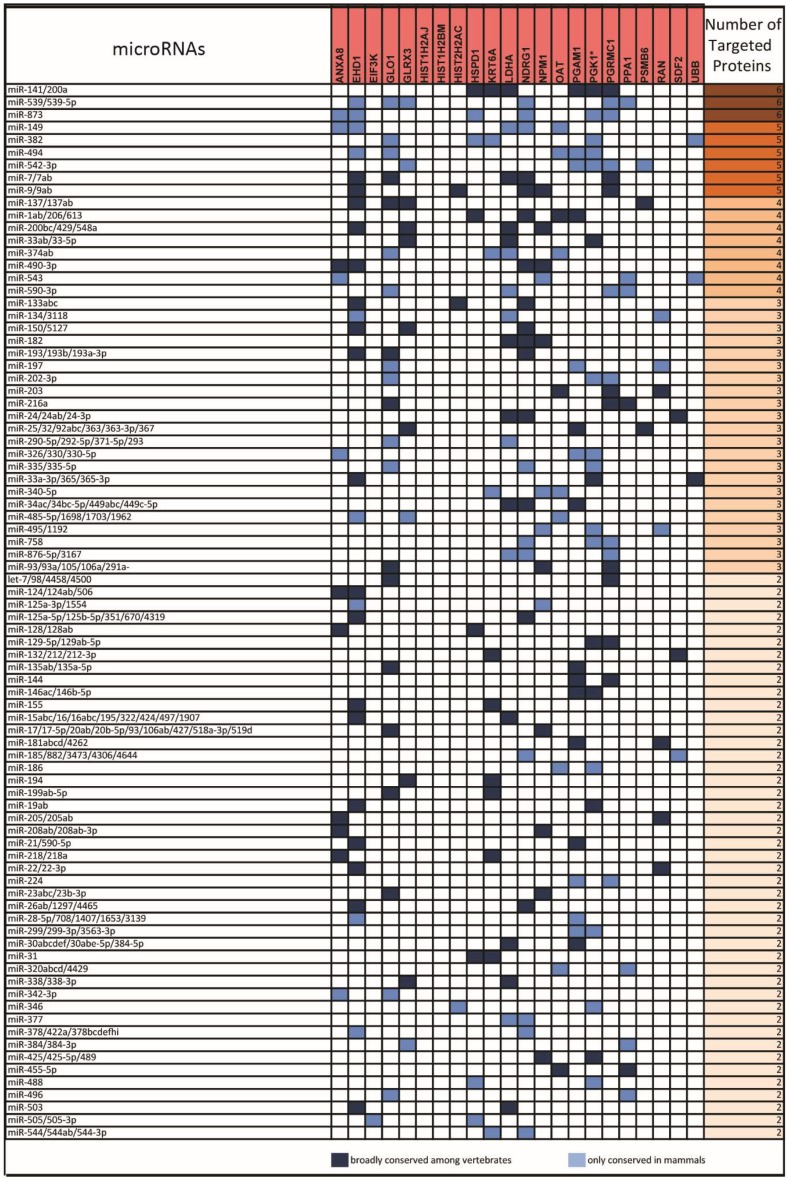
List of microRNAs (left column)-targeting the sites present in the 3′UTR of mRNA of upregulated proteins (middle column) in HME1 “DCIS-precursor” cell lines. Only target sites present in the 3’UTR of mRNAs of two or more upregulated proteins are shown (right column).

Based on this analysis, we obtained a list of 81 miRNA-target sites present in the mRNAs of two or more downregulated proteins (Figure 3) and a list of 84 sites present in the mRNAs of two or more upregulated proteins (Figure 4). 

**Figure 5:**
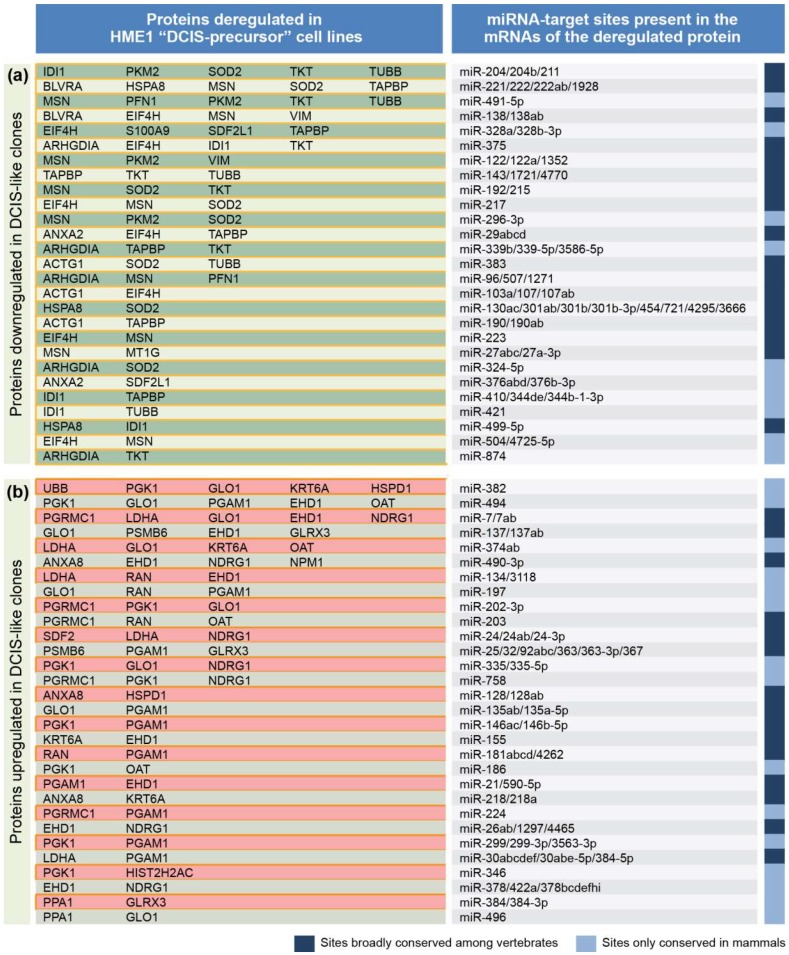
List of miRNAs based on Figure 2, including over fifty miRNAs targeting mRNAs of two or more downregulated proteins (**a**) and over fifty miRNAs targeting mRNAs of two or more upregulated proteins (**b**) in HME1 “DCIS-precursor” cells.

From the analysis in Figure 2, we identified miRNAs that target 27 sites only present in the mRNAs of downregulated proteins, and miRNAs that target 30 sites only present in the mRNAs of upregulated proteins. These miRNAs, listed in Figure 5, represent a cohort of candidate miRNAs likely deregulated in HME1 “DCIS-precursor” cell lines.

### 2.2. miRNAs Potentially Relevant to Mammary Epithelial Cell Morphogenesis

**Figure 6:**
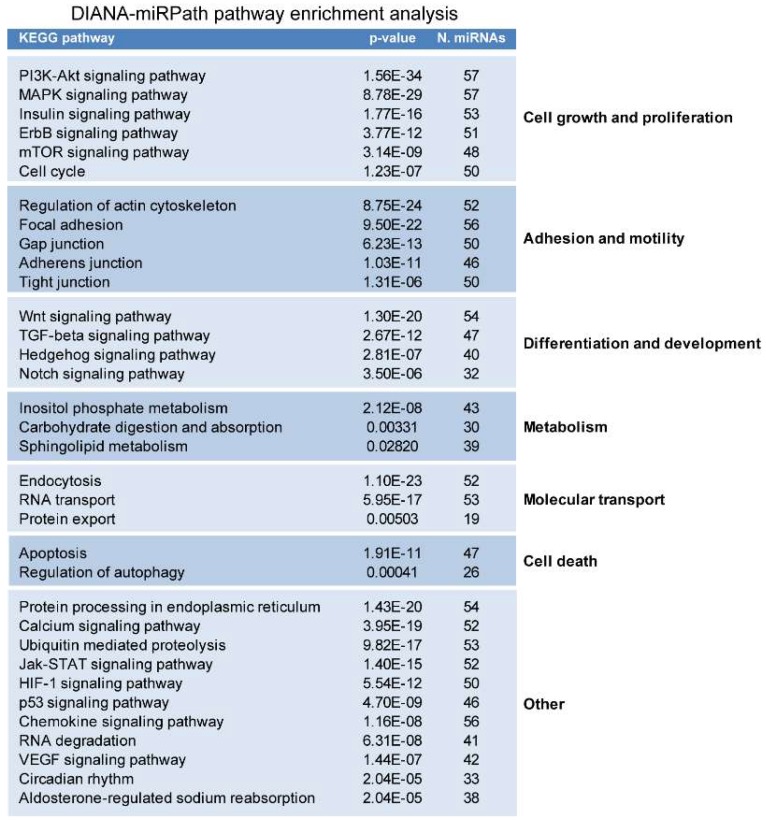
DIANA-miR Path analysis of miRNAs targeting the mRNAs of proteins deregulated in the HME1 “DCIS-precursor” cell lines shows an enrichment of cellular functions and pathways relevant to mammary epithelial morphogenesis.

To assess whether the cohort of miRNAs identified by in silico analysis is implicated in morphogenetic signaling pathways, we performed KEGG pathway enrichment analysis by using DIANA-miRPath, which can simultaneously analyze the effect of multiple miRNAs on signaling pathways using miRNA targets predicted by the DIANA-micro T-CDS algorithm [28]. This analysis showed significant enrichment of pathways involved in cellular processes known to play important roles in epithelial morphogenesis, such as cell growth and proliferation, programmed cell death, adhesion and motility, differentiation and development, metabolism, and molecular transport (Figure 6).

**Figure 7:**
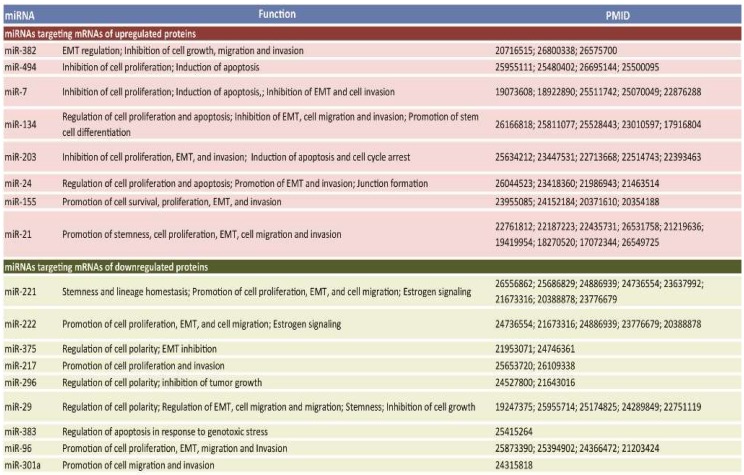
Selection of miRNAs predicted to target mRNAs of proteins deregulated in HME1 “DCIS-precursor” cell lines (left) involved in morphogenetic functions(middle), and corresponding citations identified by PMID numbers (right) found at the end of PubMed citations.

Literature mining confirmed the role of several miRNAs in these cellular processes (Figure 7). Remarkably, most of these cellular processes were also independently identified by Ingenuity Pathway Analysis (IPA) of the protein signature of the HME1 “DCIS-precursor” cell lines [24]. Apparently, the protein signature of the panel of HME1 “DCIS- precursor” cell lines could effectively predict a miRNA signature of aberrant mammary epithelial cell morphogenesis. 

### 2.3. A Subset of Deregulated miRNA in Breast Cancer

**Figure 8:**
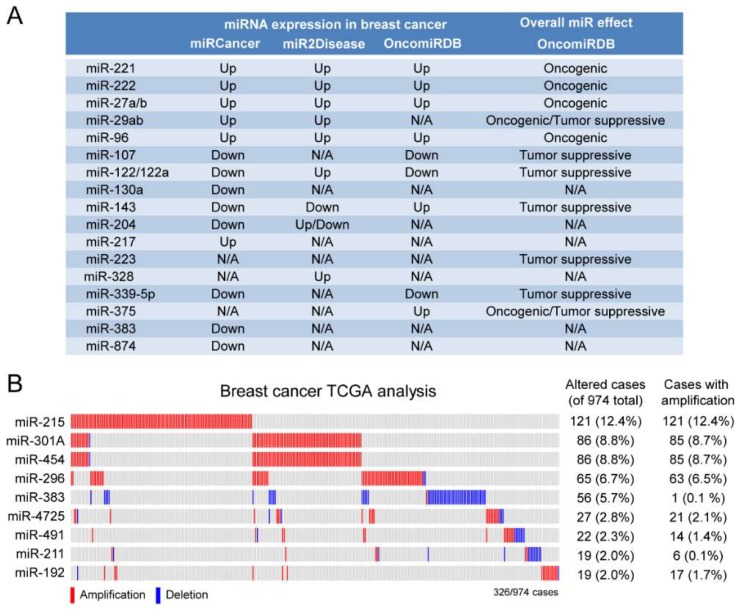
MiRNAs deregulated in breast cancer: Different miRNA databases (OncomiRDB, miRCancer, and miR2Disease) (**A**), and TCGA analysis (**B**) show that miRNAs predicted by the protein-to-miRNA approach are upregulated/amplified in breast cancer.

MiRNAs that play a role in HME1 “DCIS-precursors” might be deregulated in breast cancer and, consequently, could be potential breast cancer biomarkers. To assess whether some of these miRNAs are indeed deregulated in breast cancer we used two different approaches. First, we used OncomiRDB, miRCancer, and miR2Disease, three manually curated databases that gather published information on miRNA deregulation in disease. This combined search identified miRNAs with oncogenic properties that are found upregulated in breast cancer, including miR-221 and miR-222, which play a role in breast cancer initiation and progression by altering several oncogenic and tumor suppressor functions [29]; miR-96, which promotes breast cancer cell proliferation and invasion [30,31] and miR-27, an oncomiR whose high expression in breast cancer is associated with poor survival [32] (Figure 8A). 

Next, we expanded our analysis by interrogating The Cancer Genome Atlas (TCGA) breast cancer dataset (974 cases). As shown in Figure 8B, we found that several miRNAs were amplified in breast cancer, ranging from 2% to over 12% of cases. Among these miRNAs, we found of particular interest miR-301A and miR-454, which belong to the same miRNA family and show concomitant amplification in almost 10% of patients, and miR-215 (amplified in over 12% of cases), which was found upregulated in serum of patients with metastatic breast cancer [33]

### 2.4. An ANXA8 Feedback Loop of Aberrant Mammary Morphogenesis 

As mentioned in the introduction, ANXA8 was found upregulated in acute promyelocytic leukemia (APL) carrying the dominant negative PML-RARA mutant, that, by inhibiting the RA- regulated normal RARA transcriptional function, blocks myeloid differentiation [25], and it is associated with a restricted signature of few miRNAs (e.g., miR-342-3p), that discriminates APL myeloblasts from normal promyelocytes [23]. ANXA8 expression was also found significantly higher in DCIS relative to atypical ductal hyperplasia (ADH), and normal breast tissue, and was also associated with clinical features of breast cancer progression (e.g., positive nodes, tumor stage, and tumor grade) [24]. 

**Figure 9:**
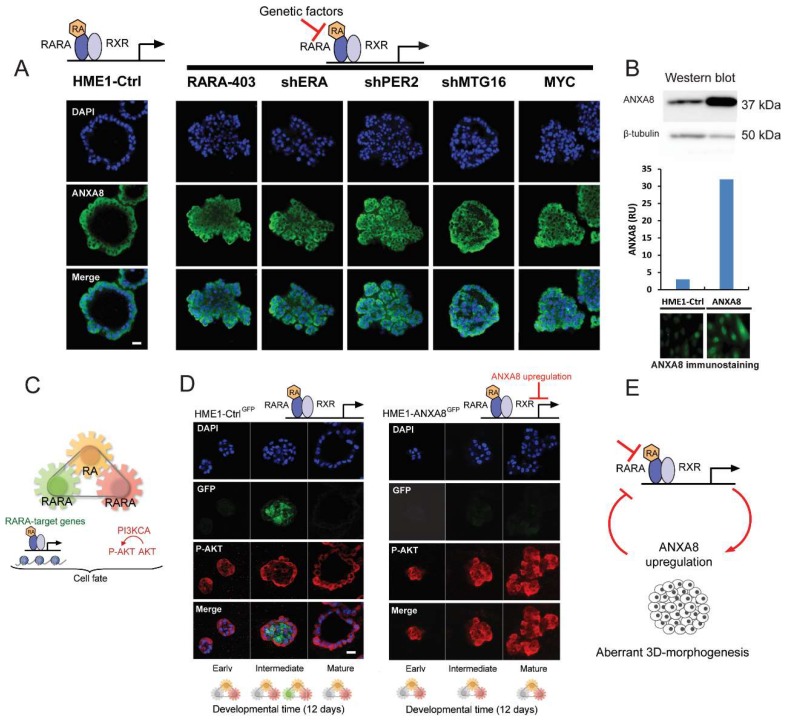
HME1-Ctrl cells with a normal RA-RARA signaling grown in reconstituted basement membrane (Matrigel) culture develop 3D ductal acinar structures with a lumen lined by ANXA8 expressing cells within 12 days (**A**, left). HME1 cells with genetic mutations that hinder RA-RARA signaling form ductal acinar structures with a lumen filled with ANXA8 expressing cells (**A**, right);scale bar: 10µm. Western blot and immunostaining show higher expression in HME1-ANXA8 cells stably expressing ectopic ANXA8 relative to HME1-Ctrl cells (**B**). Scheme of the three module mechanism whereby physiological RA (yellow) coordinates via distinct RARA functions the spatiotemporal regulation of RARA transcription (green) and PI3KCA regulation of P-AKT signaling pathway (red) (**C**), HME1-Ctrl^GFP^ cells with normal RA-RARA signaling- stably expressing a RARE-GFP construct- show P-AKT (red) positive cells at all stages of 3D acinar morphogenesis, but express only GFP (green) in luminal cells at intermediate stage (**D**, left). HME1-ANXA8^GFP^ cells show that ectopic ANXA8 expression affect the RA-regulation of RARA transcriptional function, but not the PI3K-AKT function regulated by RA, since cells are marked only by P-AKT at all stages of 3D morphogenesis (**D**, right); scale bar: 10 µm. Factors that hinder the physiological RA-RARA transcriptional mechanism by inducing ANXA8 expression reinforce a vicious circle of aberrant 3D morphogenesis (**E**).

HME1 control cells with a normal RARA/RXR transcriptional function, when grown in basement membrane culture for 12 days, form morphologically normal 3D acinar structures with a lumen lined by cells expressing endogenous ANXA8 (confocal microscopy images in Figure 9A, left). In contrast the panel of HME1 lines with mutations that affect physiological RA-RARA transcriptional function (described in the Introduction and Material and Methods) developed 3D “DCIS-like” acinar structures expressing ANXA8 in all cells (confocal microscopy images in Figure 9A, right). 

Next, we tested whether ectopic expression of ANXA8 in the HME1 cell context, was *per se* sufficient to affect 3D mammary epithelial cell morphogenesis regulated by physiological RA. To this end we developed, and characterized, HME1-ANXA8 cells stably expressing higher ANXA8 level relative to HME1-Ctrl cells by Western blot (Figure 9B, top), and immunostaining (Figure 9B, bottom). 

In normal HME1 mammary morphogenesis physiological RA coordinates in a spatiotemporal fashion two RARA functions: the canonical RARA transcriptional function, which directly regulates the chromatin state of RARA target genes, and the RARA function that regulates the activation of P-AKT via PI3KCA [13] (Scheme in Figure 9C). Both HME1-Ctrl^GFP^ cells with baseline endogenous ANXA8 expression, and HME1-ANXA8^GFP^ stably expressing ectopic ANXA8, were stably transfected with a 3x RARE-d2EGFP construct, a destabilized Green Fluorescent Protein (GFP) with a half-life of 2 h. 

In the course of 3D HME1-Ctrl^GFP^ morphogenesis we detected P-AKT (red) in cells at all stages of maturation, which indicates an active RARA-PI3KCA signaling. At intermediate stages we detected GFP expression (green) in cells destined to clear the luminal space (Figure 9D, left). In contrast, in the course of 3D HME1-ANXA8^GFP^ aberrant morphogenesis we detected P-AKT (red) in cells at all stages of acinar maturation (Figure 9D, right). These findings imply that stable ectopic ANXA8 upregulation is *per se* sufficient to inhibit the physiological RA-RARA transcriptional function, but not the physiological RA-RARA function that regulates the activation of PI3KCA- AKT signaling pathway Based on these mechanistic studies, it seems that factors (e.g., genetic mutations) that hinder the physiological RA-RARA transcriptional mechanism increase ANXA8 expression that, in turn, reinforces a vicious circle of aberrant morphogenesis (Figure 9E). 

**Figure 10:**
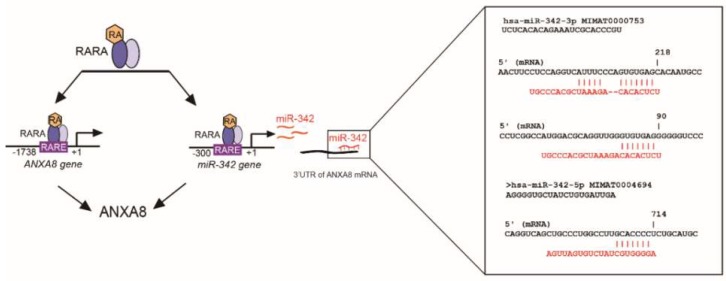
Scheme showing that ANXA8 can be regulated by RA-RARA either directly, at the ANXA8 gene promoter, or indirectly via miRNAs targeting ANXA8 mRNA 3’UTR, as the miR-342, affected in breast cancer.

As discussed hereafter not only genetic mutations affecting RA-RARA-ANXA8 feedback loop, but also other factors, as RA-regulated ANXA8 regulatory miRNAs (Figure 4 and Figure 10), might be involved in the regulation of ANXA8 during 3D mammary morphogenesis. 

## 3. Discussion

While most studies focus on identifying biomarkers in specific subset of early stage breast cancer, we use 3D HME1 DCIS models to identify regulatory molecular mechanisms and potential biomarkers and druggable targets of breast DCIS. 

In previous studies we found a restricted protein signature of 42 proteins including 22 upregulated proteins shared by five HME1 “DCIS-precursor” lines with different genetic mutations that increased the expression of ANXA8, a Ca^2+^ and phospholipid binding protein, which is regulated by all-trans Retinoic Acid (RA) [24]. ANXA8 upregulation was found upregulated for the first time in acute promyelocytic leukemia (APL) with repressed wild-type RARA transcriptional function due to dominant negative RARA fusion proteins as PML-RARA [25,26]. Breast cancer is not characterized by RARA structural mutations. However, factors that negatively affect the RARA transcriptional function predispose mammary epithelial cells to survive and proliferate thanks to the physiological RA-RARA activation of PI3KCA that affects its effectors as AKT [13]. 

Using a large panel of DCIS we found that breast DCIS tissue samples express higher ANXA8 relative to atypical ductal hyperplasia, and normal breast tissue. Moreover we found that high ANXA8 expression is also associated with clinical features of breast cancer progression (e.g., positive nodes, tumor stage, and tumor grade) [24]. In HME1 cells with wild type RARA and endogenous ANXA8 level, physiological RA exerts the spatiotemporal control of both the RARA transcriptional function and the RARA function that regulates PI3K-P-AKT signaling. HME1 cells develop 3D normal ductal structures thanks to the activation of the RARA transcriptional function in cells that are destined to clear the lumen. Cells lining the lumen are marked by P-AKT and ANXA8. HME1 “DCIS precursor cell lines” develop into 3D morphologically aberrant ductal structures lacking a lumen; all cells are marked by P-AKT and ANXA8. Consistently, ectopic expression of ANXA8 in HME1 cells, stably expressing RARE-GFP (HME1-ANXA8^GFP^), is *per se* capable of hampering the physiological RARA transcriptional function indispensable for lumen formation, but does not affect the induction of P-AKT due to RA-RARA induced PI3KCA-AKT activation. The formation of HME1 amorphous structures where ANXA8 upregulation induces P-AKT is consistent with clinical studies showing that breast DCIS are marked by high P-AKT level [34] and, as mentioned above, also by high ANXA8 [24].

By bioinformatics analysis of the protein signature of both upregulated and downregulated proteins in HME1 “DCIS-precursor” lines, we detected a restricted cohort of protein-miRNA pairs including eleven ANXA8-miRNA pairs. RA responsive elements (RAREs) are present both in the promoter of ANXA8 and in the promoters of miRNAs that target the 3’UTR of ANXA8 mRNA, including miR-128 and miR-218 that we previously described [24], and miR-342 [26,35] shown in Figure 10. MiR-218 was found downregulated in DCIS ([24] and references within), while miR-342 was found downregulated in triple negative breast cancer [35]). Downregulated miR-342 was also associated with worse overall survival in breast cancer patients [36]. 

ANXA8 upregulation in different DCIS contexts may be due to either genetic mutations affecting RA-RARA transcriptional function as shown in this study, but also to epigenetic factors like RA-regulated ANXA8 regulatory miRNAs. Additional 3D HME1 models need to be developed to assess the effects of these miRNAs on the spatiotemporal regulation of the two RARA functions, and *vice versa*.

From the translation standpoint, ANXA8 and its regulatory miRNAs might be developed as biomarkers of liquid biopsies. So far we found higher ANXA8 in exosomes released in the culture medium of HME1 cells overexpressing ANXA8 relatively to control HME1 (unpublished observations). ANXA8 might also be a potential druggable target as it is for Annexin2 (ANXA2). Recently, it was reported the identification of a new peptide that binds to and inhibits ANXA2 [37]. We previously found an inverse correlation between ANXA8 (upregulated) and ANXA2 (downregulated) in HME1-“DCIS precursor” lines. It would be interesting to identify a peptide that binds to and inhibits ANXA8. Ultimately, we hope that protein-miRNA pairs that we identified in this study might elucidate mammary additional epithelial cell morphogenetic mechanisms, and might be developed as biomarkers or druggable targets of early stage breast cancer. 

## 4. Materials and Methods 

### 4.1. Cells and Cell Culture

h-TERT-HME1 human mammary epithelial cells (here referred to as HME1) (Clontech, Mountain View, CA, USA) were grown in MEGM (Lonza, Walkersville, MD, USA) as per vendor’s instructions. The HME1-derived stable clonal lines (in this study often referred to as HME1 “DCIS-precursor” cell lines) include cell lines that we developed for different studies: HME1-RARA403 expressing a dominant negative RARA-403 mutant [14]; HME1-shERA with Estrogen Receptor alpha downregulation [15]; HME1-shPER2 with down regulation of PER2 [15]; HME1*-*shMTG16 with downregulation of the tumor suppressor MTG16 [20]; HME1-MYC overexpressing MYC oncogene [22]; HME1-ANXA8 expressing Annexin A8 [24]; HME1-ANXA8 carrying a RARE-GFP (this manuscript). Cells in 3D were cultured on growth-factor reduced Matrigel (BD Biosciences, San Jose, CA, USA) as we previously described [20]. Briefly, 3 × 10^3^ single cells/well were seeded in 8 well chamber slides on a layer of Matrigel covered with MEGM + 2% Matrigel. 3D acini were let grow for 12 days, refreshing the medium every 2–3 days. 

### 4.2. miRNA Prediction from Protein Changes

The proteomics data used for miRNA prediction were generated in [24]. For this analysis, Total ion score CI > 95% and/or protein score CI > 95 % were considered significant. Fold changes for each protein in each “DCIS-precursor” cell line were reported in a previous study of our laboratory, which is referred hereafter [24]. Messenger RNAs of the proteins deregulated in the HME1 “DCIS-precursor” cell lines were analyzed with TargetScan release 6.2 (http://www.targetscan.org/) for the presence of conserved miRNA-target sites in their 3′UTR [38]. When multiple mRNA splice variants with different 3′UTRs were identified for a single protein, all 3′UTRs were taken into consideration. The miRNA-target sites identified by TargetScan were filtered through a series of selections. First, we selected only sites broadly conserved among vertebrates or sites conserved only mammals. Second, we selected the conserved sites present in the mRNA of two or more deregulated proteins. Third, we focused on sites that were only present either in the mRNAs of proteins upregulated in the HME1 “DCIS-precursor” cell lines or in the mRNAs of proteins downregulated in the HME1 “DCIS-precursor” cell lines. After these selections, we obtained a list of miRNAs predicted to uniquely target highly conserved sited in two or more downregulated proteins or two or more upregulated proteins. This miRNA list was used for pathway analysis, database mining and TCGA analysis as described below.

### 4.3. miRNA Pathway Enrichment Analysis

We used default settings (*p* < 0.05, micro-T threshold = 0.8, gene intersection = 4, FDR= conservative) to obtain a list of pathways potentially deregulated by the miRNA listed in Figure 5. In DIANA miRPath, the P-value for each pathway is calculated by applying Fisher’s combined probability method (Fisher’s method) [28]. MicroRNA symbols obtained from TargetScan analysis were first converted into “hsa-miR-3p/5p” symbols by using miRSystem (http://mirsystem.cgm.ntu.edu.tw/) [39,40], then analyzed for KEGG pathway enrichment by using DIANA miRPath (http://diana.imis.athena-innovation.gr/DianaTools/index.php?r=mirpath/index) [28] with default settings. Significant Pathways (*p* < 0.05) were grouped according to their functions.

### 4.4. miRNA Database Mining

OncomiRDB (http://bioinfo.au.tsinghua.edu.cn/member/jgu/oncomirdb/) [41], miRCancer (http://mircancer.ecu.edu/) [42], and miR2Disease (http://www.mir2disease.org/) [43] were used to find evidence of miRNA deregulation in breast cancer. The three databases were manually searched for each miRNA individually. MiRNAs present in one or more databases were taken into consideration.

### 4.5. TCGA Analysis

The Cancer Genome Atlas (TCGA) breast invasive carcinoma dataset (TCGA) [44], was analyzed through cBioPortal for Cancer Genomics (http://www.cbioportal.org/public-portal/) [45,46]. The analysis was performed with default settings on the following genomic profiles: mutations, putative copy-number alterations from GISTIC, and a user-defined gene set containing the miRNAs listed in Figure 5. Only miRNAs altered in ≥ 2% of cases are shown in this study.

### 4.6. In Silico Identification of RARE in miRNA-342 Promoter and Sites in the ANXA8 3′UTR mRNA 

The miRNA-342 promoter region was searched for RARE sequences by using SnapGene as previously described [24]. Identification of miRNA-342 targeting sites in the ANXA8 3′UTR ([14]) was performed by using both TargetScan 6.2 release (http://www.targetscan.org/) and miRmap (https://mirmap.ezlab.org) [47]. 

### 4.7. Western Blotting (WB)

Cells were lysed with RIPA buffer (50 mM Tris-HCl pH 8.0, 150 mM NaCl, 0.1% SDS, 1% Nonidet P40, supplemented with Roche Complete protease inhibitor cocktail). Protein concentration was measured by using Coomassie Plus protein assay reagent (Thermo Scientific, Waltham, Massachusetts, USA,), and equal amounts of proteins were separated by SDS-PAGE electrophoresis and transferred onto a nitrocellulose membrane by standard methods. Membranes were incubated with anti-β-tubulin (Santa Cruz Biotechnology, Santa Cruz, CA, USA) or anti-ANXA8 (LifeSpan Biosciences, Seattle, WA, USA) washed, and incubated with an appropriate HRP-conjugated antibody (GE Healthcare, Piscataway, NJ, USA) followed by ECL detection (GE Healthcare). Protein band intensity was quantified by using Image J (NIH). 

### 4.8. Immunostaining of Normal and Aberrant 3D HME1 Acini and Confocal Analysis 

Immunostaining was performed as described [20]. Briefly, after fixation with 4% paraformaldehyde for 15 min, 3D HME1 acini were incubated with PBS + 0.2% Triton X100 for 10 min, followed by blocking with PBS + 1% BSA, 1% FBS and 0.05% Tween 20 for 1 h and incubation with the primary antibody overnight at 4 °C. Cells were then rinsed extensively with PBS, and incubated with appropriate secondary antibodies for 2 h, rinsed extensively with PBS, and stained with DAPI (Sigma, St Luis, MO, USA). Slides were mounted with Vectashield (Vector Laboratories, Burlingame, CA, USA). ANXA8 was detected with an anti-ANXA8 antibody (LifeSpan BioSciences, Seattle, WA, USA) followed by anti-rabbit Alexa Fluor 488. P-AKT was detected with an anti P-AKT (Ser 473) antibody (Cell Signaling, Danvers, MA, USA) followed by anti-rabbit Alexa Fluor 546. 3D acini were analyzed with a confocal microscope (SP2 Spectral Confocal Microscope, Leica, Wetzlar, Germany).

## 5. Conclusions

The identification of protein-miRNA pairs that are deregulated in 3D “DCIS-like” models might help us to elucidate the underpinnings of unexplored mammary morphogenetic mechanisms. So far we focused on a molecular mechanism that involves ANXA8, a member of the Annexin family, which is regulated by physiological RA via RARA. In turn, ANXA8 regulates the RA-RARA signaling, thus creating a vicious circle that fosters aberrant mammary morphogenesis. Several regulatory miRNAs might be involved in the RA-RARA-ANXA8 loop controlling 3D developmental morphogenetic processes; when deregulated these miRNAs might trigger tumorigenesis. Among other protein-miRNA pairs found in this study we expect to detect new components of mammary morphogenetic mechanisms that could be developed as DCIS biomarkers and druggable targets. 

## Abbreviations

The following abbreviations are used in this manuscript:

2D	Two dimensional
3D	Three dimensional
3’ UTR	3’ untranslated region
ANXA2	Annexin 2
ANXA8	Annexin 8
AKT	protein kinase B
DCIS	Ductal carcinoma in situ
ERA	Estrogen receptor alpha
GFP	Green Fluorescent Protein
HME1	Human mammary epithelial 1 cell line
HME1-Ctrl^GFP^	HME1 control cells carrying a stable RARE-GFP construct
HME1-RARA403	HME1 cells carrying a dominant negative RARA403 mutant
HME1-MYC	HME1 cells with MYC ectopic expression
HME1-shERA	ERA knock down HME1 cells
HME1-shMTG16	MTG16 knock down HME1 cells
HME1-shPER2	PER2 knock down HME1 cells
HME1-ANXA8	HME1 with ANXA8 ectopic expression
HME1-ANXA8^GFP^	HME1ANXA8 carrying a stable RARE-GFP construct
IPA	Ingenuity Pathway Analysis
KEGG	Kyoto Encyclopedia of Genes and Genomes
MiRNA	microRNA
PI3KCA	Phosphatidylinositol-3Kinase catalytic subunit (p110)
P-AKT	phosphorylated AKT
PMID	PubMed unique research article identifier number
RA	All- Trans Retinoic acid
RARE	RA responsive element
RARA	RA receptor alpha
TCGA	The Cancer Genome Atlas
